# Characterization of four polymorphic genes controlling red leaf colour in lettuce that have undergone disruptive selection since domestication

**DOI:** 10.1111/pbi.13213

**Published:** 2019-08-08

**Authors:** Wenqing Su, Rong Tao, Wenye Liu, Changchun Yu, Zhen Yue, Shuping He, Dean Lavelle, Weiyi Zhang, Lei Zhang, Guanghui An, Yu Zhang, Qun Hu, Robert M. Larkin, Richard W. Michelmore, Hanhui Kuang, Jiongjiong Chen

**Affiliations:** ^1^ Key Laboratory of Horticultural Plant Biology Ministry of Education Key Laboratory of Horticultural Crop Biology and Genetic improvement (Central Region) MOA College of Horticulture and Forestry Sciences Huazhong Agricultural University Wuhan China; ^2^ Genome Center and Department of Plant Sciences University of California Davis CA USA

**Keywords:** anthocyanin, leaf colour, disruptive selection, bulked segregant analysis, QTL‐seq, lettuce

## Abstract

Anthocyanins protect plants from biotic and abiotic stressors and provide great health benefits to consumers. In this study, we cloned four genes (*Red Lettuce Leaves 1* to *4: RLL1* to *RLL4*) that contribute to colour variations in lettuce. The *RLL1* gene encodes a bHLH transcription factor, and a 5‐bp deletion in some cultivars abolishes its function to activate the anthocyanin biosynthesis pathway. The *RLL2* gene encodes an R2R3‐MYB transcription factor, which was derived from a duplication followed by mutations in its promoter region. The *RLL3* gene encodes an R2‐MYB transcription factor, which down‐regulates anthocyanin biosynthesis through competing with RLL2 for interaction with RLL1; a mis‐sense mutation compromises the capacity of RLL3 to bind RLL1. The *RLL4* gene encodes a WD‐40 transcription factor, homologous to the *RUP* genes suppressing the UV‐B signal transduction pathway in Arabidopsis; a mis‐sense mutation in *rll4* attenuates its suppressing function, leading to a high concentration of anthocyanins. Sequence analysis of the *RLL1*‐*RLL4* genes from wild and cultivated lettuce showed that their function‐changing mutations occurred after domestication. The mutations in *rll1* disrupt anthocyanin biosynthesis, while the mutations in *RLL2*,* rll3* and *rll4* activate anthocyanin biosynthesis, showing disruptive selection for leaf colour during domestication of lettuce. The characterization of multiple polymorphic genes in this study provides the necessary molecular resources for the rational breeding of lettuce cultivars with distinct levels of red pigments and green cultivars with high levels of health‐promoting flavonoids.

## Introduction

Anthocyanins are naturally occurring flavonoid chromophores that are largely responsible for the variation in colour among flowers and fruits, an important adaptive trait in plants. Anthocyanins may also accumulate in leaves to protect the photosynthetic apparatus and photolabile compounds from ultraviolet radiation (Gould, 2004). The accumulation of anthocyanins may be induced by environmental stressors such as UV damage, extreme temperatures and drought (Winkel‐Shirley, 2002). Elevated levels of anthocyanins participate in abiotic or biotic stress tolerance in plants by scavenging reactive oxygen species (Winkel‐Shirley, 2002). The antioxidant activity of anthocyanins also provides great health benefits to consumers (Liobikas *et al*., 2016; Morais *et al*., 2016; Qin *et al*., 2018).

The flavonoid biosynthetic pathway in plants has been well studied. Flavonoid biosynthetic genes can be divided into early biosynthetic genes (EBGs), which catalyse the production of dihydroflavonols and late biosynthetic genes (LBGs), which lead to the biosynthesis of proanthocyanidins and anthocyanins (Ferreyra *et al*., 2012; Xu *et al*., 2015). The flavonoid biosynthetic pathway is mainly regulated by a complex of three transcription factors: MYB, bHLH and WD40 (MBW) (Hichri *et al*., 2011). The MBW complex is regulated by several factors (Li, 2014). In Arabidopsis, MYBL2 negatively regulates the MBW complex by competing with R2R3‐MYBs for interactions with the bHLH component of the MBW complex (Dubos *et al*., 2008; Matsui *et al*., 2008). SPL9, a target of miR156/157, disrupts the MBW complex by competitively binding to the R2R3‐MYB subunit of the MBW complex (Gou *et al*., 2011). TCP3 interacts with R2R3‐MYB proteins and promotes flavonoid biosynthesis (Li and Zachgo, 2013). The RUP1 and RUP2 proteins are two WD40‐repeat proteins that serve as negative regulators of UV‐B signalling and anthocyanin biosynthesis by down‐regulating the expression of *HY5*, which regulates the expression of genes in the anthocyanin biosynthesis pathway (Gruber *et al*., 2010). The R2R3‐MYB subunit of the MBW complex may also be regulated by other proteins such as nitrate‐responsive proteins and RING E3 ligase (An *et al*., 2017; Gruber *et al*., 2010; Wang *et al*., 2018b). The expression of genes associated with anthocyanin biosynthesis is often regulated by biotic and abiotic stress and by environmental factors, such as light and temperature.

Genetic cloning of genes responsible for the natural variation in anthocyanin levels in crops may provide novel insights into the regulation of anthocyanin biosynthesis in diverse plants and will provide tools that are directly applicable to breeding programs. High‐throughput sequencing technology has revolutionized the genetic analysis of important traits in crops. The combination of bulked segregant analysis (BSA) and high‐throughput sequencing has been proven to be an efficient and cost‐effective method for the genetic analysis of important traits in crops (Dou *et al*., 2018; Huo *et al*., 2016; Michelmore *et al*., 1991). It can be also used for genetic analysis of QTLs, which was termed QTL‐seq (Illa‐Berenguer *et al*., 2015; Lu *et al*., 2014; Singh *et al*., 2016). Another powerful genetic approach is the genome‐wide association study (GWAS), which has been used to study QTLs controlling important traits in many crops (Bazakos *et al*., 2017).

Lettuce (*Lactuca sativa*) is one of the most important vegetable crops worldwide. Lettuce genomics has advanced rapidly in recent years (Reyes‐Chin‐Wo *et al*., 2017; Zhang *et al*., 2017). Cultivated lettuce shows dramatic variations in morphology and leaf colour. Previous studies on the inheritance of anthocyanin in lettuce suggested several loci controlling anthocyanin accumulation (Robinson *et al*., 1983). In our previous GWAS, we identified 12 expression quantitative trait loci (eQTL) that regulate the expression of 24 genes associated with flavonoid biosynthesis (Zhang *et al*., 2017) and several candidate loci controlling the accumulation of anthocyanins in lettuce leaves (Zhang *et al*., 2017). However, functional analysis of these candidate genes on the accumulation of anthocyanins was beyond the scope of our initial study. In addition, their evolution and their impact on the regulatory mechanisms that drive the accumulation of anthocyanins remained to be studied.

In this report, we used BSA + RNA‐seq to determine the genetics underlying the red colour of lettuce leaves. Four genes controlling leaf colour in lettuce were genetically mapped, cloned and characterized. We studied the impact of these genes on the mechanisms that regulate the accumulation of anthocyanins in lettuce. The evolution and applied significance of these genes controlling leaf colour of lettuce are discussed.

## Results

### Colour variation in wild and domesticated lettuce

**Figure 1:**
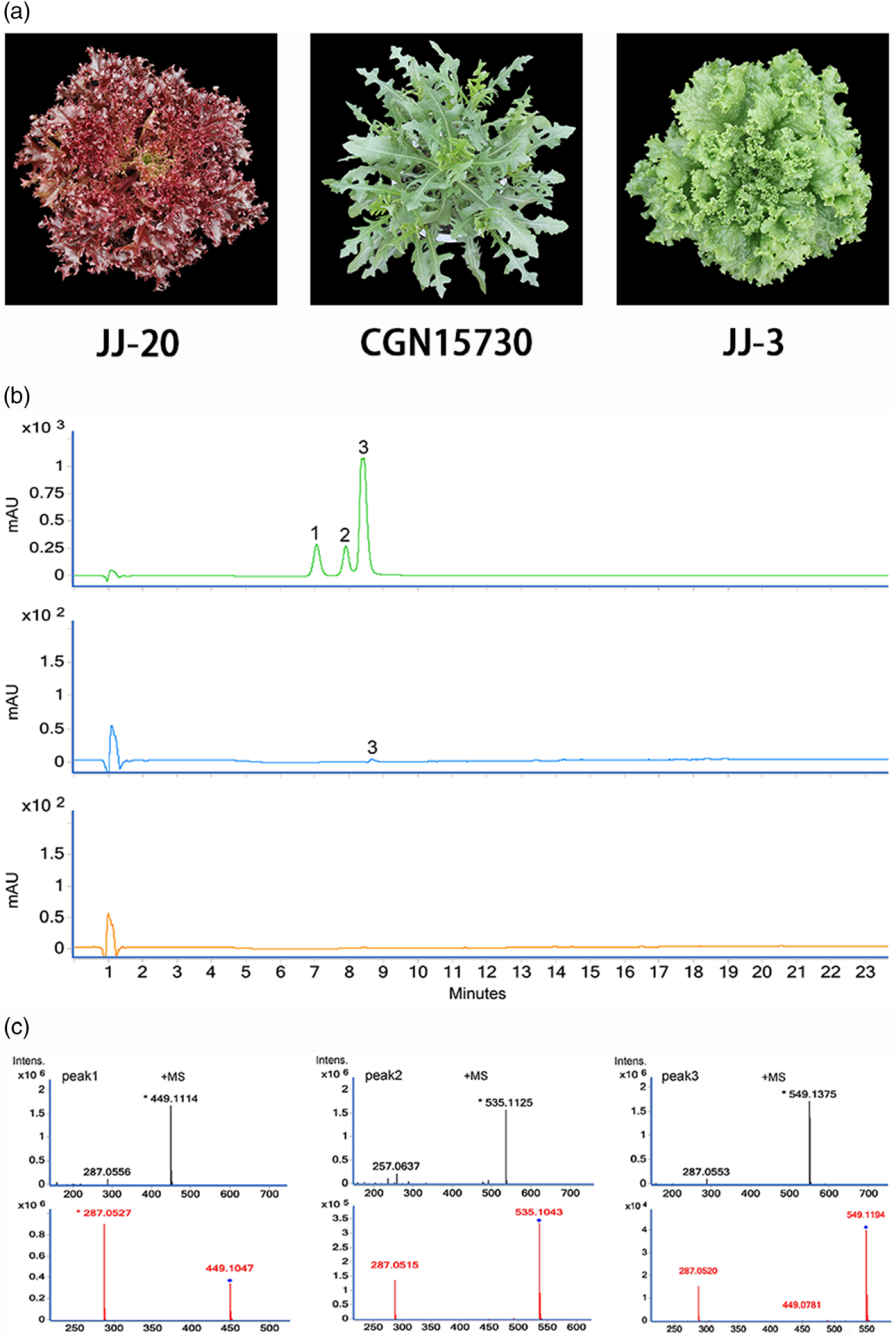
Disruptive selection of anthocyanin accumulation in lettuce. (a) Leaf colour of a red cultivar (left), a wild lettuce (middle) and a green cultivar (right). (b) HPLC analysis of methanolic extracts from the red cultivar (top), the wild genotype (middle) and the green cultivar (bottom). HPLC chromatograms were recorded at 525 nm. Each chromatogram was generated using identical quantities of injected samples. (c) LC‐MS profile of anthocyanins extracted from the red cultivar. Peak 1: cyanidin‐3‐*O*‐(6″‐malonyl‐β‐glucopyranoside); Peak 2: cyanidin‐3‐*O*‐β‐glucopyranoside; and Peak 3: cyanidin‐3‐*O*‐(6″‐malonyl‐β‐glucopyranoside methyl ester).

The leaf colours of 240 lettuce genotypes used previously for GWAS in Zhang *et al*. (2017) were investigated (Zhang *et al*., 2017). We also expanded the sampling to include 123 additional wild lettuce genotypes, which typically have green leaves, from different parts of the world (Table S1). In total, 145 genotypes were investigated in this study, including the 22 genotypes from the GWAS population. After treatment with cold or drought, the leaves of wild lettuce may, to some extent, appear red. Unlike wild lettuce, some cultivars do not accumulate red pigments in response to stress. However, other cultivars of lettuce may develop light to dark red leaves when grown under non‐stress conditions. A red cultivar, a green cultivar and a wild progenitor of lettuce were randomly chosen to investigate the types and relative concentrations of anthocyanins (Figure 1a). Using HPLC‐tandem mass spectrometry (MS/MS), three peaks were identified in red cultivars (Figure 1c). Peaks 1, 2 and 3 correspond to three cyanidin derivatives: cyanidin‐3‐*O*‐(6″‐malonyl‐β‐glucopyranoside), cyanidin‐3‐*O*‐(6″‐malonyl‐β‐glucopyranoside methyl ester) and cyanidin‐3‐*O*‐β‐glucopyranoside, respectively (Mulabagal *et al*., 2010). We quantified the relative levels of anthocyanins in the different genotypes using HPLC (Figure 1b). The green cultivar has no detectable anthocyanin, the red cultivar has abundant anthocyanin, while the wild progenitor accumulated intermediate levels of anthocyanins.

### QTL‐seq evidence for multiple loci controlling the accumulation of anthocyanins in lettuce

To identify loci that affect the accumulation of anthocyanins in lettuce, a lettuce cultivar (S1, with dark red leaves) was crossed with a cultivar of stem lettuce (Y37, with green leaves). The F_1_ population was selfed to generate a segregating F_2_ population. Among 218 F_2_ individuals, 113 plants had green leaves and the other 105 developed pigmentation phenotypes that ranged from light red to dark red. This pattern of phenotypic segregation suggests that the accumulation of anthocyanins in leaves is controlled by both qualitative and quantitative polymorphic loci in this population.

**Figure 2:**
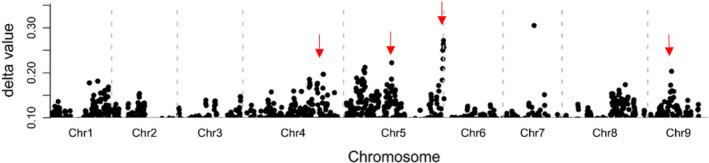
Differences in allele frequencies between the red and green pools from the F2 population. The *x*‐axis represents the nine chromosomes of lettuce. The *y*‐axis indicates the difference in allele frequencies (SNP values) between the red pool and the green pool. Multiple peaks of SNP values are shown along the nine chromosomes, suggesting several loci contributing to the variation in leaf colour variation in the segregating population. The positions of the four genes that we cloned from this population are marked with arrows. 84 × 24 mm.

From this F_2_ population, 50 plants with the most intense red colour were chosen for a ‘red pool’ and 50 plants with green leaves were randomly chosen for a ‘green pool’. Equal amounts of leaf tissue were collected from each individual and pooled. RNA was extracted from each contrasting pool of leaves. The RNA‐seq libraries were sequenced, and reads were mapped to the lettuce genome sequence v8 (Reyes‐Chin‐Wo *et al*., 2017). Single nucleotide polymorphisms (SNPs) were detected, and allele frequencies for these SNPs were calculated for each pool. The difference in the allele frequency (index) between the two pools was calculated for each SNP and was plotted along the nine chromosomes (Figure 2). The plot figure exhibited multiple peaks of SNP values. These data provide evidence that several loci contributed to the variation in leaf colour in this F_2_ population. To verify these loci, markers were designed at four loci and were used to screen the F_2_ population (Table S2). Two loci, *Red Lettuce Leaf 1* (*RLL1*) and *Red Lettuce Leaf 2* (*RLL2*), were shown to be statistically associated with variations in leaf colour (i.e. green or red leaves) in this population. F_2_ individuals that are homozygous for the Y37 allele at the *RLL1* locus (i.e. *rll1*/*rll1*) always have green leaves. Similarly, most F_2_ individuals that are homozygous for the Y37 allele at the *RLL2* locus (i.e. *rll2*/*rll2*) have green leaves. The combination of the *RLL1* and *RLL2* loci explained the colour variation (i.e. green or red leaves) for the majority of individuals in the F_*2*_ population. Based on these data, we concluded that *RLL1* and *RLL2* are two qualitative loci.

### The *RLL1* gene encodes a bHLH transcription factor

**Figure 3:**
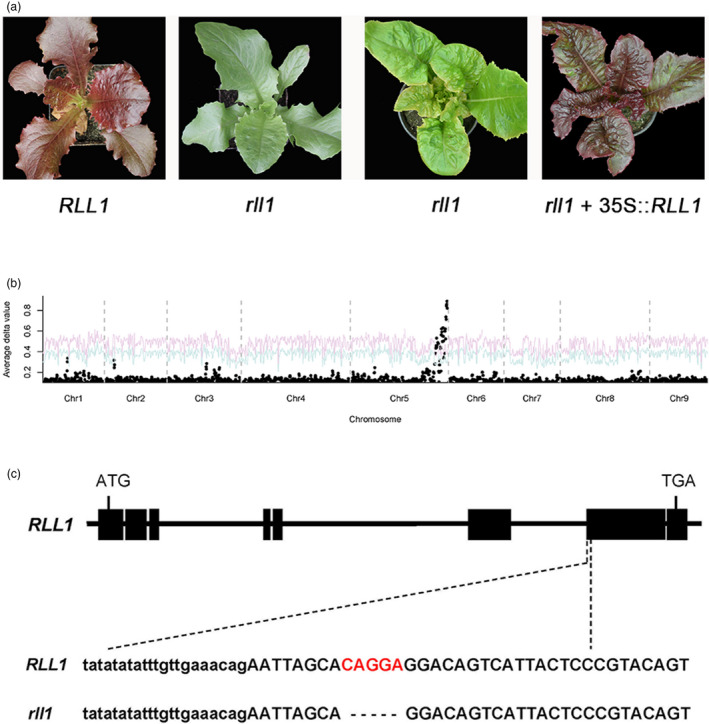
Cloning of the RLL1 gene. (a) The leaf colour of RLL1 and rll1 in the segregating population (left panel), and a green individual (rll1 genotype) from the segregating population and its transgenic rll1 + 35S::RLL1 (right panel). (b) Mapping of the RLL1 gene using BSA. The red and blue curves represent confidence probability of *P* = 0.01 and *P* = 0.05, respectively. (c) Structure of the RLL1 gene. The black boxes represent exons, and the lines between the boxes represent introns. The 5‐bp deletion in rll1 is indicated below the relevant exon.

To fine map and clone the *RLL1* gene, F_*2*_ individuals with *RLL1/rll1* and *RLL2/RLL2* genotypes were selfed to generate F_2:3_ families. One F_2:3_ family (S9) with red to green phenotypes segregating in an approximately 3:1 ratio was chosen for fine mapping of the *RLL1* gene (Figure 3a). RNA‐seq analysis of a red pool and a green pool from the S9 F_2:3_ family confirmed that the leaf colour was controlled by a single locus on chromosome 5. We named this locus *Red Lettuce Leaves 1* (*RLL1*) (Figure 3b). Using 1751 green individuals (chosen from an F_2:3_ family of 5100 individuals), the *RLL1* gene was mapped between 335.69 and 337.92 Mb on chromosome 5 (Table S2). This interval contains a gene that encodes a putative bHLH transcription factor. Based on a GWAS and a gene expression network analysis, this gene had been predicted to be associated with the accumulation of anthocyanins (Zhang *et al*., 2017). Comparison of gene sequences of the *bHLH* gene from the two parents revealed a 5‐bp deletion at the beginning of exon 7 in the green parent that is not present in the red parent (Figure 3c). This 5‐bp deletion in the green parent causes a frameshift and consequently a null allele (*rll1*). Transformation of a green individual from the segregating population with the wild‐type *RLL1* gene from the red parent (S1) yielded transgenic progeny with red leaves. These data indicate that this bHLH‐encoding gene is *RLL1* and that *RLL1* promotes the accumulation of anthocyanins in lettuce (Figure 3a).

### The *RLL1* gene up‐regulates the expression of anthocyanin biosynthetic pathway genes

The RNA‐seq data from the red and green pools that were prepared from the S9 F_2:3_ family indicated that four differentially expressed genes (DEGs) are associated with anthocyanin biosynthesis (*CHS*,* DFR*,* ANS* and *GST*) in this subpopulation. The differences in their expression were confirmed with a qRT‐PCR analysis that utilized near‐isogenic lines (NILs) that differed in the genomic region containing the *RLL1* and *rll1* alleles (Figure S1A). Y1H assays demonstrated that the RLL1 transcription factor binds the promoters of the *DFR* and *ANS* genes (Figure S1B). Therefore, the loss‐of‐function mutation in the bHLH‐encoding gene (i.e. *rll1*) down‐regulates the expression of multiple genes in the biosynthetic pathway of anthocyanin and leads to constitutively green leaves.

### *RLL2* encodes a MYB transcription factor

**Figure 4:**
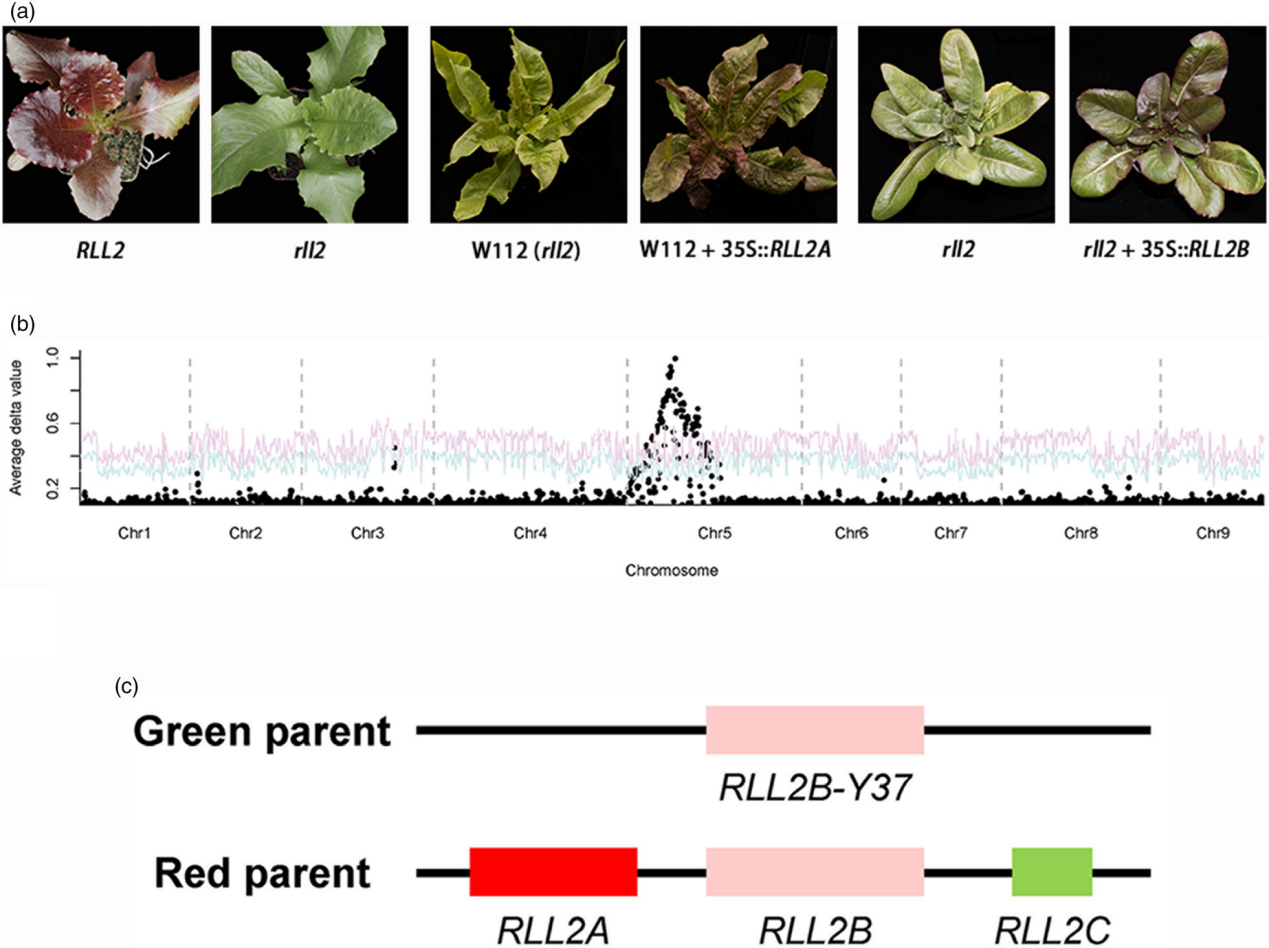
Cloning of the RLL2 gene. (a) Leaf colour of RLL2 and rll2 in the segregating population (left panel), rll2 and rll2 + 35S::RLL2A (middle panel) and rll2 and rll2 + 35S::RLL2B (right panel). Note that the three rll2 genotypes in the three panels have different genetic backgrounds. (b) Mapping of the RLL2 gene using BSA. The red and blue curves represent confidence probability of *P* = 0.01 and *P* = 0.05, respectively. (c) Haplotypes of the RLL2 locus in the green and red parents. The relative positions of the three copies in the red parent are undetermined.

A F_2:3_ family (Q16) that was homozygous for *RLL1* (from the red parent) and that was segregating the *RLL2* locus was used to clone the *RLL2* gene. The Q16 F_2:3_ family had 4058 red and 1372 green individuals (Figure 4a), with a phenotypic ratio of approximately 3:1 (*P* > *0.5*). A BSA + RNA‐seq experiment confirmed that a single locus on chromosome 5 was responsible for the segregating leaf colour in this family (Figure 4b). By genotyping the green individuals (recessive homozygotes), the *RLL2* gene was mapped to a 2.6‐Mb region on chromosome 5 (Figure S2A and Table S2). Based on a GWAS and a gene expression network analysis, a gene encoding a MYB transcription factor from this locus was predicted to be associated with the accumulation of anthocyanins (Zhang *et al*., 2017). To test this hypothesis, we PCR amplified the homologs of this MYB‐encoding gene from the red (S1) and the green parents (Y37). Three homologs (*RLL2A*,* RLL2B* and *RLL2C*) were amplified from the genome of the red parent, which were all mapped to the same locus using gene‐specific markers (Figure 4c). In contrast, one homolog (*RLL2B‐Y37*) was found in the genome of the green parent, which is identical to the gene in the reference genome. Only the *RLL2A* gene from the red parent is highly expressed (Figure S2B). Transformation of the green genotype with the *RLL2A* gene from the red parent yielded transgenic lines with red leaves (Figure 4a). These data demonstrate that the *RLL2A* copy is the *RLL2* gene, which promotes the accumulation of anthocyanins in lettuce.

### Members of the *RLL2* gene family regulate anthocyanin biosynthesis

Both the *RLL2B* and *RLL2B‐Y37* alleles were expressed at low levels and therefore probably do not contribute to the observed colour variation in the Q16 family. To test whether the *RLL2B* gene can regulate anthocyanin biosynthesis when expressed, the coding sequences of *RLL2B* driven by the 35S promoter were transformed into a green genotype from the segregating population, and the green phenotype successfully changed to red phenotype. Therefore, when expressed at elevated levels, *RLL2B* can promote the accumulation of anthocyanins.

From an RNA‐seq analysis of the red and green pools from the Q16 family, we identified 93 DEGs, including four from the anthocyanin biosynthesis pathway (*F3H, DFR, ANS* and *AAC*). The differential expression of these four genes was confirmed using qRT‐PCR (Figure S2B). Y1H assays indicated that the RLL2 protein binds the promoters of the *DFR* and *ANS* genes (Figure S2C). Therefore, the RLL2 protein appears to promote the accumulation of anthocyanins by directly binding the promoters of genes that encode anthocyanin biosynthetic enzymes. Y1H assays demonstrated that the RLL2B proteins could also bind to the promoter of the *ANS* and *DFR* genes, which is consistent with the overexpression of *RLL2B* promoting the accumulation of anthocyanin (Figure S2C).

### *RLL3* encodes a MYB transcription factor and repressor of anthocyanin biosynthesis

**Figure 5:**
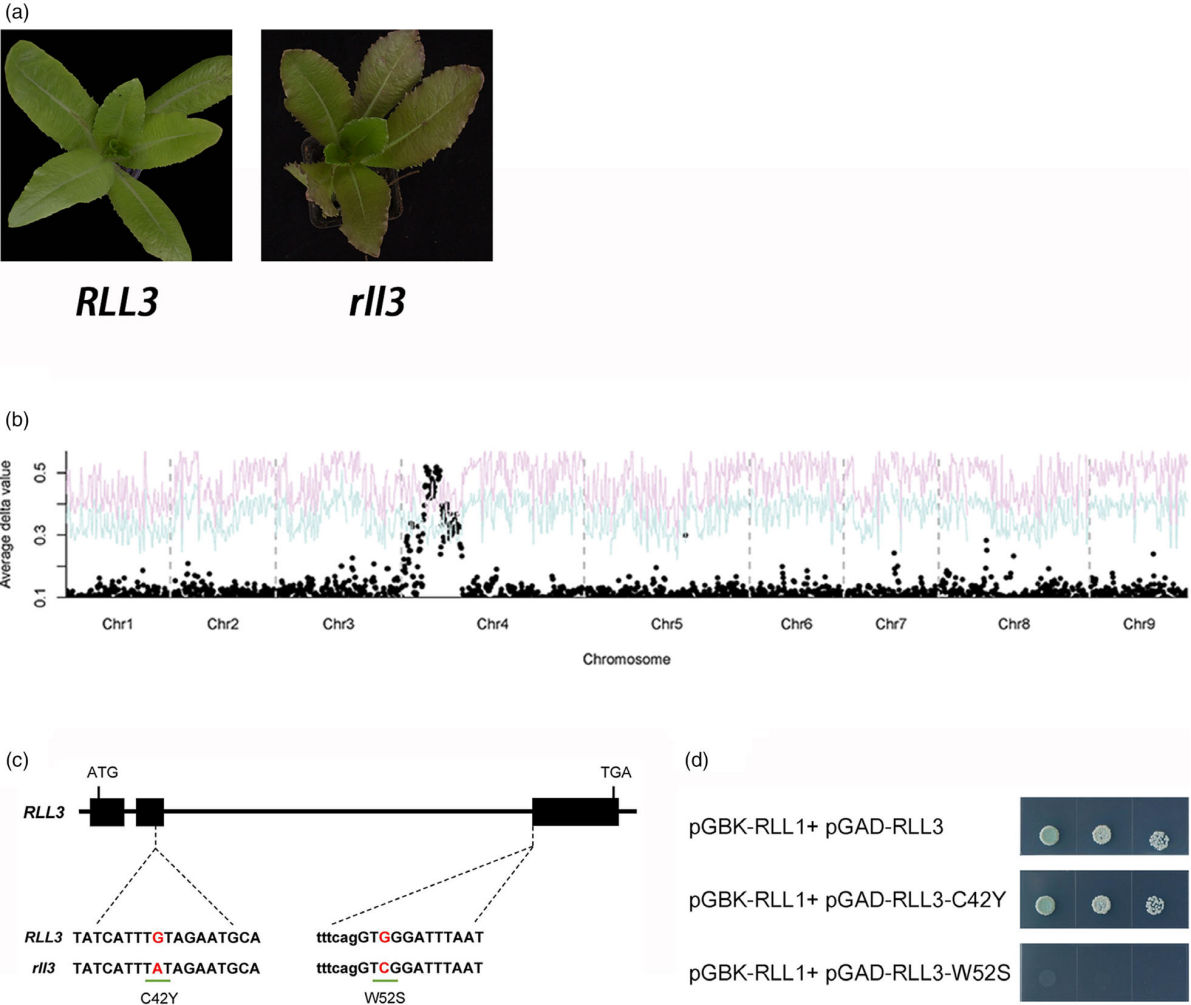
Cloning of the RLL3 gene. (a) Leaf colour of RLL3 and rll3 in the segregating population. (b) Mapping of the RLL3 gene using BSA. The red and blue curves represent confidence probability of *P* = 0.01 and *P* = 0.05, respectively. (c) Gene structure of the RLL3 gene. The black boxes represent exons. The lines between the boxes represent introns. The two SNPs are shown. (d) Yeast two‐hybrid assays. These data indicate that RLL1 and RLL3 can interact and that the W52S substitution in RLL3 but not the C42Y substitution in RLL3 compromised these interactions.

Although most individuals with *RLL1*/*RLL1*_*rll2*/*rll2* (null allele) genotypes have green leaves, some individuals became light red when the seedlings were approximately 1 month old (Figure 5a). Thus, we hypothesized that there is an additional locus that promotes the accumulation of anthocyanins that partially rescues the *rll2* green phenotype. We chose an F_2:3_ family with a *RLL1*/*RLL1*_*rll2*/*rll2* genetic background that segregated green to light red phenotypes in a 3:1 ratio to study this locus controlling leaf colour. A BSA + RNA‐seq analysis demonstrated that the colour variation in this subpopulation was controlled by a single locus on chromosome 4 that we named *RLL3* (Figure 5b). However, we originally failed to independently confirm this result with the F_2_ population due to interference from *RLL1* and *RLL2*. Our previous GWAS also identified a significant locus in this region and gene expression network analysis suggested *CAD* as a candidate gene (Zhang *et al*., 2017). This locus contributed a moderate peak of SNP values to our earlier BSA + RNA‐seq experiment (Figure 2). However, using a large segregating population (864 recessive homozygotes with red leaves), the *CAD* gene was excluded as the candidate gene. Instead, the 500‐kb candidate region contains a gene encoding a R3‐MYB transcription factor. Two SNPs (G/A and G/C) in the CDS of this *MYB* gene cause amino acid substitutions (C42Y and W52S) (Figure 5c).

### RLL3 negatively regulates anthocyanin biosynthesis by competing with RLL2 for a binding site on RLL1

The predicted RLL3 protein has the conserved [D/E]Lx2[R/K]x3Lx6Lx3R motif in the R3 domain and belongs to the R3‐MYB group of MYB transcription factors. The results from our Y1H experiments indicate that RLL3 does not bind the promoters of the genes that contribute to anthocyanin biosynthesis, probably due to the lack of a DNA‐binding domain in the C‐terminus. We hypothesized that the R3‐MYB RLL3 protein negatively regulates anthocyanin biosynthesis by competing with the RLL2 protein for a binding site on the bHLH subunit of the MBW complex (Baudry *et al*., 2004).

Y2H assays were used to test the above hypothesis. Y2H showed that RLL1 (bHLH) interacted with RLL2 (R2R3‐MYB) and LsTTG1, the WD40 component in the MBW complex (Walker *et al*., 1999) (Figure 5d). Interestingly, RLL1 also interacted with the negative regulator RLL3 (R3‐MYB). This interaction was abolished by the W52S substitution present in the rll3 protein (Figure 5d). These results are consistent with our hypothesis that RLL3 suppresses anthocyanin biosynthesis by competing with RLL2 for a binding site on RLL1. Additionally, these data are in accordance with the amino acid substitution in rll3 abolishing this suppression and consequently promoting the accumulation of anthocyanins in the leaves of *rll3*.

### *RLL4* is homologous to the *RUP* genes in Arabidopsis

**Figure 6:**
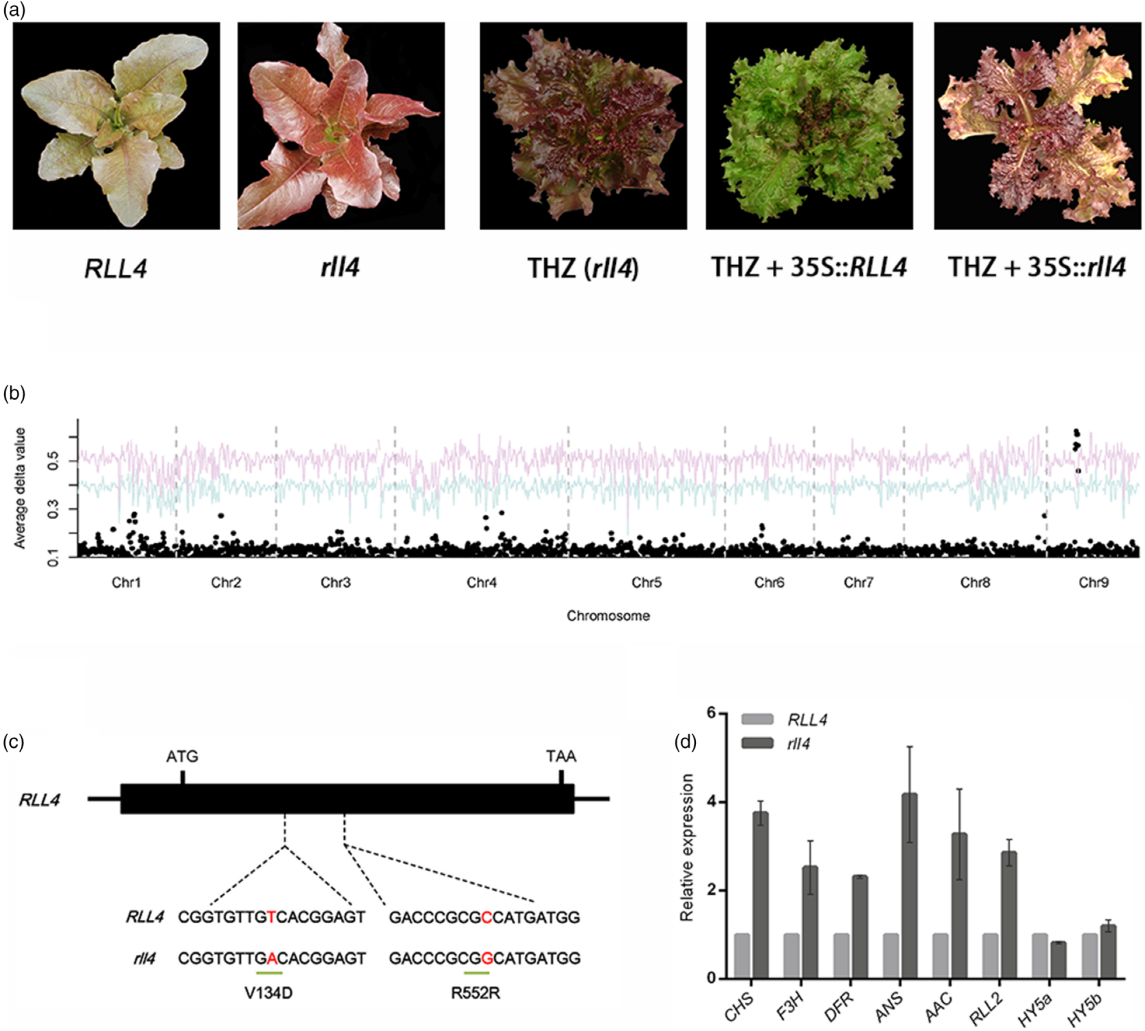
Cloning of the RLL4 gene. (a) Leaf colour of plants in the segregating population (left panel), red cultivar THZ (rll4) and transgenic plants with 35S::RLL4 and 35S::rll4 (right panel). (b) Mapping of the RLL4 gene using BSA. A NIL was used as the mapping population. The red and blue curves represent confidence probability of *P* = 0.01 and *P* = 0.05, respectively. (c) Structure of the RLL4 gene. The black boxes represent exons, and the lines between the boxes represent introns. The two SNPs are shown. (d) Expression of anthocyanin‐associated genes in the RLL4 and rll4 genotypes of NIL. Gene expression was quantified using qRT‐PCR. The expression of RLL2 and several genes required for anthocyanin biosynthesis was down‐regulated in RLL4 relative to rll4. RLL4 did not affect the expression of HY5a or HY5b. Data are means ± SEM (*n* = 3 biological replicates).

Mutations in *RLL1*,* RLL2* and *RLL3* did not explain all of the colour variation in the F_2_ population, indicating the presence of an additional locus/loci contributing to the colour variation in lettuce. To test this idea, we planted 114 F_4_ families and found that leaf colour segregated in 32 of these families. The colour variation in the 32 F_4_ families was screened using markers derived from *RLL1*,* RLL2* and *RLL3* (Table S2). One F_4_ family, Q54, was homozygous at all three loci but still segregated light red and dark red leaves at a ratio of 195:75 (light red leaves to dark red leaves), which provided evidence for an additional locus (*RLL4*) controlling leaf colour (Figure 6a). We mapped this gene to chromosome 9 using BSA + RNA‐seq (Figure 6b) and used 3100 individuals to fine map this gene to a 358‐kb region containing five candidate genes (Table S2). One of these genes encodes a WD40 transcription factor. Bi‐direction BLAST and phylogenetic analyses indicated that this WD40‐encoding gene is an ortholog of the *RUP1* and *RUP2* genes in Arabidopsis, which are negative regulators of UV‐B signalling and anthocyanin biosynthesis (Gruber *et al*., 2010) (Figure S3). Transgenics with the 35S promoter driving the expression of this *RLL4* candidate gene had considerably attenuated the red colour in leaves, which confirmed that the *RLL4* gene suppresses the accumulation of anthocyanins (Figure 6a).

### Point mutation at the conserved WD40 domain‐encoding region abolishes the function of RLL4, leading to up‐regulation of flavonoid biosynthesis genes

Unexpectedly, the *RLL4* gene was expressed at slightly higher levels in the dark red individuals than in the light red individuals, which appears to conflict with its function as a suppressor of anthocyanin accumulation. We speculated that its sequence rather than its expression level was responsible for the variation in leaf colour in the segregating population. Sequence comparisons showed two SNPs between *RLL4* and *rll4*. One of them results in an amino acid change (V134D) in the conserved WD40 domain (Figure 6c). Overexpression of the mutated *rll4* had no obvious phenotypic change, which is consistent with the above hypothesis that the point mutation in the WD40 domain‐encoding region rather than its expression level determines the functional variations of the *RLL4* alleles (Figure 6a).

A qRT‐PCR analysis demonstrated that *RLL4* significantly down‐regulated the expression of anthocyanin biosynthesis genes such as *RLL2*,* CHS*,* F3H*,* DFR, ANS* and *AAC* in the NILs (Figure 6d). The down‐regulation of anthocyanin biosynthesis genes by *RLL4* orthologs (*RUP1* and *RUP2*) was also found in Arabidopsis (Gruber *et al*., 2010). This down‐regulation functions through the *HY5* gene, which connects the UV‐B pathway and the anthocyanin pathway (Brown *et al*., 2005; Gruber *et al*., 2010). Unlike in Arabidopsis, the expression of the two *HY5* genes in lettuce (*HY5a* and *HY5b*) was not affected by the *RLL4* gene (Figure 6d), suggesting an alternative regulatory mechanism of *RLL4* on anthocyanin biosynthesis in lettuce.

### Genetic deficiencies that prevent the accumulation of anthocyanins were selected at an early stage of domestication

To understand the evolution of the four genes cloned in this study, their sequences were obtained from a panel of *Lactuca* genotypes that includes 124 cultivars from all horticultural types and 145 wild genotypes. The 5‐bp deletion in the *RLL1* gene, which abolished its function, was found in 32 cultivars including both leafy and stem lettuce. All cultivars with this *rll1* allele have green leaves. On the other hand, all of the 145 wild genotypes collected from all over the world have the wild‐type *RLL1* allele. Based on these data, we conclude that the loss‐of‐function mutation in the *RLL1* gene occurred in an ancient cultivated population of lettuce.

### The red colour of lettuce leaves was selected after domestication

We also investigated the evolution of the three genes that promote the accumulation of anthocyanins in lettuce (*RLL2*,* RLL3* and *RLL4*). The *RLL3* gene is a negative regulator of anthocyanin biosynthesis. The mis‐sense mutation in *RLL3* that is responsible for the pigmentation phenotype was not found in the wild genotypes, but was found in three cultivars of loose leaf, eight romaine, three butterhead and four crisphead. Similarly, the causal mis‐sense mutation responsible for the V134A substitution in *RLL4* was found only in five cultivated genotypes. Therefore, the loss‐of‐function mutations in the *RLL3* and *RLL4* genes that promote the accumulation of anthocyanins in lettuce leaves were selected after domestication.

**Figure 7:**
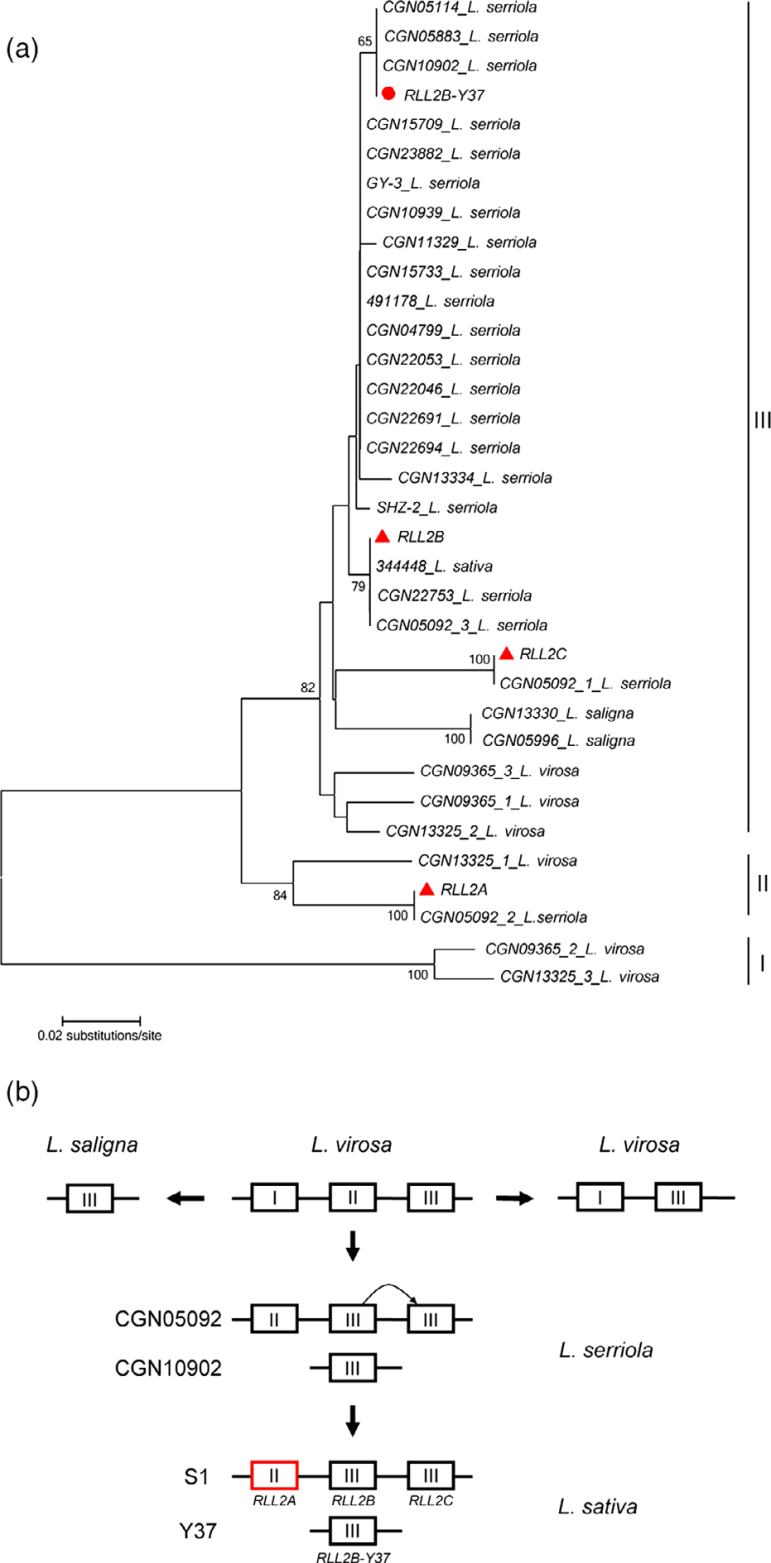
Evolution of the RLL2 gene. (a) Distance tree of the RLL2 homologs from different Lactuca species. A neighbour‐joining (NJ) tree was constructed for RLL2 using DNA sequences containing part of exon II, part of exon III and all of intron II. The three genes from S1 are marked by red triangles, and RLL2B_Y37 is indicated by a circle. Bootstrap values are percentages from 1000 replicates. Values lower than 65 are not shown. The RLL2 homologs amplified from an accession are named with the accession number followed by the species name. (b) The evolution of the RLL2 locus in Lactuca. I, II and III refer to the three clades in (a). The mutation at the promoter of the gene (in red) in clade II in some cultivars led to its accelerated expression. Represented genotypes are listed on the left.

To investigate the evolution of the *RLL2* gene family in *Lactuca*, PCR primers were designed at conserved regions and used to amplify ~700‐bp PCR products from 124 cultivars and 145 wild genotypes of lettuce (Table S2). The PCR products were sequenced directly; 228 of the 269 genotypes have a single homolog of *RLL2* that could be classified into 21 distinct groups (with at least one SNP between two groups). The other 41 genotypes have two or more homologs of *RLL2* that could be grouped into three distinct *RLL2* haplotypes. Genotypes CGN05092, CGN09365 and CGN13325 representing the three haplotypes were chosen to characterize their *RLL2* homologs. TA cloning and subsequent sequencing discovered three *RLL2* homologs from CGN05092, CGN09365 and CGN13325, respectively. Consequently, a total of 34 distinct *RLL2* genes were obtained from the 269 genotypes and a phylogenetic tree was constructed (Figure 7a).

The sequences of the approximately 700‐bp fragments from 39 of the 269 genotypes are identical to the *RLL2* sequences, including two genotypes of *L. serriola* (CGN05092 and CGN11333). Further analysis showed that these two wild genotypes have the *RLL2* haplotype similar to that of the S1 parent, that is with three copies of *RLL2* homologs (*RLL2_CGN05092* and *RLL2_CGN11333*). However, the expression of the *RLL2_CGN05092* gene was significantly lower than that of the *RLL2* gene in S1. We obtained the genomic sequences of *RLL2_CGN05092*, including 1.43 kb of sequences in the promoter region. The difference between *RLL2* and *RLL2_CGN05092* is a 2‐bp (TA) insertion in *RLL2* at −411 bp and three SNPs in the coding region. It is most likely that the 2‐bp insertion in the promoter of the *RLL2* gene from S1 activates the gene, resulting in red leaves. Therefore, spontaneous mutations in the *RLL2* gene might have been selected after domestication (Figure 7b).

## Discussion

Anthocyanin in lettuce is one of the earliest Mendelian traits studied in plants (Dahlgren, 1918; Durst, 1915). In this study, we identified five polymorphic genes controlling anthocyanin accumulation in lettuce. Based on the description of the inheritance and phenotypes, the *C* and *T* genes investigated by Thompson 80 years ago are likely the *RLL1* genes characterized in this study, because (i) *RLL1* is complementary to each other and (ii) homozygotes of loss‐of‐function mutation for either of them completely block anthocyanin biosynthesis in lettuce, which were also the characteristics of the *C* and *T* genes (Thompson, 1938). The *i* gene reported by Lindqvist (1960) resembles the *RLL3* or *RLL4* gene in this study, since they are recessive intensifiers (Lindqvist, 1960). The *R* locus studied by Thompson (1938) is very likely to be the *RLL2* locus, which controls the intensity of the pigment and spots on lettuce leaves (Thompson, 1938).

### Application of BSA + high‐throughput sequencing to study complex genetic traits

Bulked segregant analysis is a fast, economical and efficient method for genetic analysis (Michelmore *et al*., 1991). High‐throughput sequencing technology is now frequently used with BSA to provide large numbers of informative markers (Ding *et al*., 2017; Dou *et al*., 2018; Wang *et al*., 2018a). In this study, we used BSA + RNA‐seq to efficiently analyse the genetics of red leaf colour in lettuce. Two qualitative loci (*RLL1* and *RLL2*) were easily identified through an analysis of allele frequencies in two contrasting pools. Due to several factors, the other two loci (*RLL3* and *RLL4*), which are QTLs, were not detected in our initial analysis. First, the two qualitative loci are epistatic to the two quantitative loci, and consequently, the differences in the allele frequencies of the two quantitative loci were below detection limits. Second, although a large number of individuals (50) in the green pool may reduce sampling error, a large number of individuals in the red pool may decrease the frequencies of the two colour‐promoting quantitative loci. Furthermore, if the original F_2_ population had been larger, we would have identified the two quantitative loci using only the dark red individuals for the red pool. Furthermore, due to the epistatic effects of *RLL1* and *RLL2*, we needed to use only red individuals rather than the entire F_2_ population to identify *RLL3* and *RLL4*. Since the biosynthetic pathway and the regulatory network that drives the accumulation of anthocyanins are highly conserved in plants, homology searches in combination with expression profiling analysis are commonly used to identify genes that are associated with the accumulation of flavonoids in non‐model species, such as lettuce (Zhang *et al*., 2016). On the other hand, the genetic dissection of the natural variation of anthocyanin levels in crops will show its underlying genetic events, which may provide novel insight into anthocyanin biosynthesis and the regulatory factors that drive the accumulation of anthocyanins, such as the case for the *RLL4* gene in this study. This information may be valuable to future breeding programs.

### Negative regulators of anthocyanin biosynthesis

Loss‐of‐function mutations in any gene that contributes to anthocyanin biosynthesis may attenuate the accumulation of anthocyanins. For example, a mutation in the *ANS* gene characterized in this study blocks the conversion of leucoanthocyanidin, a colourless flavonoid, to downstream coloured products. Furthermore, loss‐of‐function alleles can arise from different types of mutations, such as indels that cause frameshifts (Quattrocchio *et al*., 1999; Tang *et al*., 2017). Therefore, mutations that attenuate the accumulation of anthocyanins may occur frequently in nature. In contrast, gain‐of‐function mutations are relatively rare. Moreover, a gain‐of‐function mutation in a single gene will only affect the accumulation of the final products of a particular biosynthetic pathway (e.g. the anthocyanin biosynthetic pathway) if the gain‐of‐function mutation affects a rate‐limiting step in the pathway. Transcription factors, such as R2R3‐MYB transcription factors, regulate entire pathways. Thus, we expect that gain‐of‐function mutations in genes encoding transcription factors more commonly promote high‐level accumulation of final products than genes encoding biosynthetic enzymes. In this study, we discovered that high‐level expression of a MYB transcription factor leads to the high‐level accumulation of anthocyanins in lettuce leaves. Similar results were reported in cauliflower, orange and rice (Butelli *et al*., 2012; Chiu *et al*., 2010; Oikawa *et al*., 2015).

Negative regulators of anthocyanin biosynthesis have been reported in plants, such as the *RUP1* and *RUP2* genes and the *R3‐MYB* genes (Dubos *et al*., 2008; Gruber *et al*., 2010; Matsui *et al*., 2008) (Aharoni *et al*., 2001; Cao *et al*., 2017). As mentioned above, spontaneous loss‐of‐function mutations should occur at a much higher rate than spontaneous gain‐of‐function mutations. Interestingly, we discovered loss‐of‐function mutations in the orthologs of a *R3‐MYB* gene and the *RUP1* and *RUP2* genes in lettuce (i.e. *RLL3* and *RLL4*). These mutations considerably increased the abundance of anthocyanins in several cultivars.

The role of *RLL4* in anthocyanin biosynthesis in lettuce is highly similar to that of *RUP1* and *RUP2* in Arabidopsis, since their loss‐of‐function mutations up‐regulate the expression of anthocyanin‐associated genes. However, in contrast to Arabidopsis, *RLL4* in lettuce does not regulate anthocyanin biosynthesis through *HY5*. Our Y2H experiments suggested no interactions between RLL4 and RLL2, therefore ruling out RLL4 as a competitor of TTG1 (the WD40 component of the MBW complex). Nevertheless, anthocyanin biosynthesis in lettuce is UV‐B dependent, and UV‐B treatment induces the expression of *HY5*. Therefore, it is most likely that *HY5* also bridges the UV‐B pathway and anthocyanin biosynthesis pathway in lettuce. Knockout of genes in the UV‐B pathway, such as *HY5* and *UVR8*, in future studies, may shed light on the regulatory mechanism of RLL4 on anthocyanin biosynthesis.

### Disruptive selection for leaf colour in lettuce

When grown in optimal conditions, wild lettuce usually develops green leaves with occasional accumulation of anthocyanins near the edge of the leaf lamina. Stress, such as drought, promotes the accumulation of anthocyanins leading to visibly red colour of wild lettuce leaves. However, some lettuce cultivars lost this stress response because of loss‐of‐function mutations in a *bHLH*‐encoding gene. On the other hand, some lettuce cultivars develop red leaves when they are grown in optimal conditions. The constitutive up‐regulation of anthocyanin biosynthesis is due to a gain‐of‐function mutation in a gene encoding a MYB transcription factor and loss‐of‐function mutations in two negative regulators. Therefore, lettuce cultivars that develop green leaves and cultivars that develop red leaves were both selected during domestication and in modern breeding programmes, showing typical artificial disruptive selection, that is selection favouring extreme values over intermediate values (Krakauer, 2018). Cultivars lacking anthocyanins may decrease their ability against biotic and abiotic stresses. On the other hand, cultivars with high concentration of anthocyanins in their epidermal cells will block the penetration of lights and consequently reduce photosynthesis and plant growth. The selection on green and/or red leaves of a vegetable has been carried out by human being, that is artificial selection. Selection on leaf colour may also exist in nature. For example, trees with bright colour of leaves may reduce their parasite load since insects tend to avoid laying their eggs on bright trees (Archetti and Brown, 2004).

As a salad vegetable, it is important for lettuce to develop diverse colours. Although anthocyanins are appreciated for their health‐promoting benefits, green lettuce is still the main type of lettuce sold on the market. The combination of the anthocyanin‐promoting alleles of four genes (*RLL1*,* RLL2*,* rll3* and *rll4*) with the mutated *ans* gene is predicted to generate a green lettuce cultivar, which accumulates abundant colourless flavonoids, providing health benefits to consumers.

## Materials and methods

### Mapping population

To study the genetics underlying leaf colour in lettuce, a loose‐leaf cultivar with red leaves (Figure 1) was crossed with a green stem cultivar (Y37). The F_1_ hybrid was selfed to generate a segregating F_2_ population of 218 plants. The association of a marker with leaf colour was tested using a chi‐square test. Each F_2_ individual was selfed for three generations. A large number of seeds were obtained for each line in each generation. These lines were screened to choose informative families for genetic mapping. The lettuce plants were grown in the National Center for Vegetable Improvement (Central China) on the campus of Huazhong Agricultural University in Wuhan, China.

### BSA RNA‐seq

For each segregating population, two pools each containing 50 individuals with contrasting phenotypes were constructed. Tissue from the young leaves of 1‐month‐old plants was pooled, and total RNA was extracted using the TRIzol reagent (Invitrogen, Paisley, UK). RNA was quantified and assessed using a Qubit Fluorometer and a Nanodrop spectrophotometer (Novogene, Beijing, China). Paired‐end sequencing was carried out on an Illumina HiSeq 2500 instrument. Raw RNA‐seq data were mapped to the lettuce genome assembly v8 (Reyes‐Chin‐Wo *et al*., 2017), and SNPs were called. The frequency of each SNP was calculated for both pools. The frequency difference (∆Index) between the two pools was calculated, and their positions on the nine lettuce chromosomes were plotted. A region with a high ∆Index potentially harboured a gene controlling the trait that was used to construct the contrasting pools. RNA‐seq data were available at GenBank under the following accession number PRJNA512330.

### Yeast one‐hybrid (Y1H)

To perform Y1H assays, the oligonucleotide sequences containing the core elements of the bHLH binding region (G‐box and E‐box) and MYB binding site (MBS) were arrayed in tandem and cloned into pHIS2, respectively. The coding region of the transcription factors was cloned into pGADT7. The yeast strain AH109 was transformed with each construct using the LiAc‐PEG method as described in the manual from the manufacturer (Clontech, Mountain view, CA, http://www.clontech.com/). The transformed yeast strains were grown on SD/‐Trp/‐Leu medium, and then, different dilutions were spotted on SD/‐Trp/‐Leu/‐His medium in the presence or absence of 45 mm 3‐aminotriazole (Sigma‐Aldrich, http://www.sigmaaldrich.com). The plates were incubated for 3 days at 28 °C. Cell growth was used to assay the activity of the reporter gene.

To test whether the RLL1 transcription factor binds the promoters of the genes encoding anthocyanin biosynthetic enzymes, the oligonucleotide sequences containing the core elements of the bHLH binding regions, the E‐box (CANNTG) and the G‐box (CACGTG) in the promoters of the anthocyanin structural genes were identified at PlantCARE (http://bioinformatics.psb.ugent.be/webtools/plantcare/html/), arrayed in tandem and cloned into pHIS2 vectors. Three truncated fragments of the RLL1 coding sequence were fused to the AD domain coding sequence to test whether RLL1 can interact with the promoter sequences of *CHS*,* CHI*,* DFR*,* F3H* and *ANS* because the *RLL1* protein had a strong autoactivation activity when fused to the GAL4 activation domain.

### Yeast two‐hybrid (Y2H)

Y2H analysis was performed using the Matchmaker Gold Yeast Two‐Hybrid Library Screening System (Clontech). The ORF of *bHLH* genes were amplified by PCR and inserted into pGBKT7. *MYB* genes were cloned into pGADT7 vector. All fusion constructs were introduced into yeast strain AH109 using the LiAc‐PEG method as described in the manual from the manufacturer (Clontech). The transformants were selected on SD/‐Leu/‐Trp medium. The positive colonies were transferred to SD/‐Leu/‐Trp/‐His/‐Ade medium containing X‐α‐Gal (5‐bromo‐4‐chloro‐3‐indolyl‐α‐d‐galactopyranoside) to test for interactions between pairs of proteins. Photographs were taken after 3–5 days of growth on the medium at 28 °C.

### Transformation and complementation tests

To transform lettuce, the full‐length cDNAs from genes of interest were amplified and cloned into pHellsgate8 (Invitrogen), pRI101 and p06 vectors driven by CaMV 35S promoter. *Agrobacterium tumefaciens* strain GV3101 was transformed with the constructs that were used for plant transformation using the freeze–thaw method. Transgenic plants were generated using cotyledon explants (Michelmore *et al*., 1987) and were selected on MS medium supplemented with 40 mg/L kanamycin or 30 mg/L hygromycin by the UC Davis Parsons Plant Transformation Facility (http://ucdptf.ucdavis.edu).

### Extraction of anthocyanins and LC‐MS analyses

Lettuce leaves were lyophilized and ground into a fine powder. A total of 0.2 g of this fine powder was extracted with 3 mL of extraction solution (CH_3_OH containing 1% HCl) at 4 °C for 16 h. The extracts were centrifuged at 1878 ***g*** at 4 °C for 10 min. The supernatants were transferred to new 2‐mL centrifuge tubes. The extracts were filtered using 0.22‐μm nylon syringe filters before being analysed by LC‐MS. Gradient elution was performed at a flow rate of 0.3 mL/min with solvent A (1% aqueous acetic acid) and solvent B (1% acetic acid and 95% acetonitrile) as follows: 0–20 min, 5%–95% B; 20–24 min, 95% B; 24–26 min, 95%–5% B; and 26–30 min, 5% B. The positive ion mode (m/z M+H^+^) was used to detect the anthocyanins.

### Sequence analysis

*RLL2* homologs were amplified using primers that annealed to conserved sequences. The PCR products were cloned into *pEASY*‐T5 Zero vector (TransGen Biotech, Beijing, China). For the last five clones that were sequenced, individual clones were sequenced until no new sequence data were obtained. Sequences were aligned using Geneious (Drummond, 2010), and phylogenetic trees were constructed using MEGA7.0 (Kumar *et al*., 2016).

### Quantitative RT‐PCR analysis

RNA was extracted using TRIzol reagent (Invitrogen). Approximately 2 μg of total RNA per sample was used to synthesize first‐strand cDNA using TransScript One‐Step gDNA Removal and cDNA Synthesis SuperMix (TransGen Biotech). Quantitative RT‐PCR was performed using SYBR premix Ex Taq (ABMgood, Vancouver, Canada) and the CFX96 Touch System (Bio‐Rad, Hercules, CA). The amplification programme was performed at 95 °C for 30 s, followed by 95 °C for 1 s and 60 °C for 10 s (40 cycles). Ubiquitin was used to normalize the qPCR data.

### Accession numbers

RNA‐seq data are available at GenBank under the following accession number PRJNA512330, and gene sequences have accession numbers of MK522155–MK522161.

## Author contributions

J.C. and H.K. designed the project. W.S., R.T., W.L., C.Y., Z.Y., S.H., D. L. and G.A. performed the experiments. W.Z., L.Z., Y.Z., Q.H. and D. L. analysed the experimental data. J.C., W.S and R.T. wrote the manuscript with the help of H.K., R.M.L. and R.W.M.

## Conflict of interest

The authors declare no conflict of interests.

## Supporting information


**Figure S1** Characterization of *RLL1*.
**Figure S2** Mapping and functional analysis of the *RLL2* gene.
**Figure S3** Phylogenetic analysis of RLL4 and its homologs.
**Table S1** Summary of the 145 wild lettuce accessions.
**Table S2** Primers used in this study.

## References

[pbi13213-bib-0001] Aharoni, A. , De Vos, C.H.R. , Wein, M. , Sun, Z.K. , Greco, R. , Kroon, A. , Mol, J.N.M. *et al*. (2001) The strawberry *FaMYB1* transcription factor suppresses anthocyanin and flavonol accumulation in transgenic tobacco. Plant J. 28, 319–332. 11722774 10.1046/j.1365-313x.2001.01154.x

[pbi13213-bib-0002] An, J.P. , Liu, X. , Li, H.H. , You, C.X. , Wang, X.F. and Hao, Y.J. (2017) Apple RING E3 ligase MdMIEL1 inhibits anthocyanin accumulation by ubiquitinating and degrading MdMYB1 protein. Plant Cell Physiol. 58, 1953–1962. 29016961 10.1093/pcp/pcx129

[pbi13213-bib-0003] Archetti, M. and Brown, S.P. (2004) The coevolution theory of autumn colours. Proc. Biol. Sci. 271, 1219–1923. 15306345 10.1098/rspb.2004.2728 PMC1691721

[pbi13213-bib-0004] Baudry, A. , Heim, M.A. , Dubreucq, B. , Caboche, M. , Weisshaar, B. and Lepiniec, L. (2004) TT2, TT8, and TTG1 synergistically specify the expression of *BANYULS* and proanthocyanidin biosynthesis in *Arabidopsis thaliana* . Plant J. 39, 366–380. 15255866 10.1111/j.1365-313X.2004.02138.x

[pbi13213-bib-0005] Bazakos, C. , Hanemian, M. , Trontin, C. , Jimenez‐Gomez, J.M. and Loudet, O. (2017) New strategies and tools in quantitative genetics: how to go from the phenotype to the genotype. Annu. Rev. Plant Biol. 68, 435–455. 28226236 10.1146/annurev-arplant-042916-040820

[pbi13213-bib-0006] Brown, B.A. , Cloix, C. , Jiang, G.H. , Kaiserli, E. , Herzyk, P. , Kliebenstein, D.J. and Jenkins, G.I. (2005) A UV‐B‐specific signaling component orchestrates plant UV protection. Proc. Natl Acad. Sci. USA, 102, 18225–18230. 16330762 10.1073/pnas.0507187102 PMC1312397

[pbi13213-bib-0007] Butelli, E. , Licciardello, C. , Zhang, Y. , Liu, J.J. , Mackay, S. , Bailey, P. , Reforgiato‐Recupero, G. *et al*. (2012) Retrotransposons control fruit‐specific, cold‐dependent accumulation of anthocyanins in blood oranges. Plant Cell, 24, 1242–1255. 22427337 10.1105/tpc.111.095232 PMC3336134

[pbi13213-bib-0008] Cao, X. , Qiu, Z.K. , Wang, X.T. , Van Giang, T. , Liu, X.L. , Wang, J. , Wang, X.X. *et al*. (2017) A putative *R3 MYB* repressor is the candidate gene underlying *atroviolacium*, a locus for anthocyanin pigmentation in tomato fruit. J. Exp. Bot. 68, 5745–5758. 29186488 10.1093/jxb/erx382 PMC5854135

[pbi13213-bib-0009] Chiu, L.W. , Zhou, X.J. , Burke, S. , Wu, X.L. , Prior, R.L. and Li, L. (2010) The purple cauliflower arises from activation of a MYB transcription factor. Plant Physiol. 154, 1470–1480. 20855520 10.1104/pp.110.164160 PMC2971621

[pbi13213-bib-0010] Dahlgren, K.V.O. (1918) Über einige Kreuzungsversuche mit *Chelidonium majus* L., *Polemonium coeruleum* L. und *Lactuca muralis* L. Svensk. Bot. Tidskrift, 12, 103–110.

[pbi13213-bib-0011] Ding, B.Q. , Mou, F.J. , Sun, W. , Chen, S.L. , Peng, F. , Bradshaw, H.D. and Yuan, Y.W. (2017) A dominant – negative actin mutation alters corolla tube width and pollinator visitation in *Mimulus lewisii* . New Phytol. 213, 1936–1944. 28164332 10.1111/nph.14281 PMC5300067

[pbi13213-bib-0012] Dou, J.L. , Zhao, S.J. , Lu, X.Q. , He, N. , Zhang, L. , Ali, A. , Kuang, H.H. *et al*. (2018) Genetic mapping reveals a candidate gene (*ClFS1*) for fruit shape in watermelon (*Citrullus lanatus* L.). Theor. Appl. Genet. 131, 947–958. 29362832 10.1007/s00122-018-3050-5

[pbi13213-bib-0013] Drummond, A.J. , e.a. (2010) Geneious v4.8. Available from http://www.geneious.com

[pbi13213-bib-0014] Dubos, C. , Le Gourrierec, J. , Baudry, A. , Huep, G. , Lanet, E. , Debeaujon, I. , Routaboul, J.M. *et al*. (2008) MYBL2 is a new regulator of flavonoid biosynthesis in *Arabidopsis thaliana* . Plant J. 55, 940–953. 18532978 10.1111/j.1365-313X.2008.03564.x

[pbi13213-bib-0015] Durst, C.E. (1915) Studies in lettuce breeding. Proc. Amer. Soc. Hort. Sci. 12, 96–98.

[pbi13213-bib-0016] Ferreyra, M.L.F. , Rius, S.P. and Casati, P. (2012) Flavonoids: biosynthesis, biological functions, and biotechnological applications. Front. Plant Sci. 3, 222. 23060891 10.3389/fpls.2012.00222 PMC3460232

[pbi13213-bib-0017] Gou, J.Y. , Felippes, F.F. , Liu, C.J. , Weigel, D. and Wang, J.W. (2011) Negative regulation of anthocyanin biosynthesis in *Arabidopsis* by a miR156‐targeted SPL transcription factor. Plant Cell, 23, 1512–1522. 21487097 10.1105/tpc.111.084525 PMC3101539

[pbi13213-bib-0018] Gould, K.S. (2004) Nature's swiss army knife: the diverse protective roles of anthocyanins in leaves. J. Biomed. Biotechnol. 2004, 314–320. 15577195 10.1155/S1110724304406147 PMC1082902

[pbi13213-bib-0019] Gruber, H. , Heijde, M. , Heller, W. , Albert, A. , Seidlitz, H.K. and Ulm, R. (2010) Negative feedback regulation of UV‐B‐induced photomorphogenesis and stress acclimation in *Arabidopsis* . Proc. Natl Acad. Sci. USA, 107, 20132–20137. 21041653 10.1073/pnas.0914532107 PMC2993346

[pbi13213-bib-0020] Hichri, I. , Barrieu, F. , Bogs, J. , Kappel, C. , Delrot, S. and Lauvergeat, V. (2011) Recent advances in the transcriptional regulation of the flavonoid biosynthetic pathway. J. Exp. Bot. 62, 2465–2483. 21278228 10.1093/jxb/erq442

[pbi13213-bib-0021] Huo, H.Q. , Henry, I.M. , Coppoolse, E.R. , Verhoef‐Post, M. , Schut, J.W. , de Rooij, H. , Vogelaar, A. *et al*. (2016) Rapid identification of lettuce seed germination mutants by bulked segregant analysis and whole genome sequencing. Plant J. 88, 345–360. 27406937 10.1111/tpj.13267

[pbi13213-bib-0022] Illa‐Berenguer, E. , Van Houten, J. , Huang, Z. and van der Knaap, E. (2015) Rapid and reliable identification of tomato fruit weight and locule number loci by QTL‐seq. Theor. Appl. Genet. 128, 1329–1342. 25893466 10.1007/s00122-015-2509-x

[pbi13213-bib-0023] Krakauer, A. (2018). Disruptive selection. In Encyclopedia of Evolutionary Psychological Science ( Shackelford, T.K. and Weekes‐Shackelford, V.A. , eds), pp. 1–3. Cham: Springer International Publishing.

[pbi13213-bib-0024] Kumar, S. , Stecher, G. and Tamura, K. (2016) MEGA7: molecular evolutionary genetics analysis version 7.0 for bigger datasets. Mol. Biol. Evol. 33, 1870–1874. 27004904 10.1093/molbev/msw054 PMC8210823

[pbi13213-bib-0025] Li, S. (2014) Transcriptional control of flavonoid biosynthesis: fine‐tuning of the MYB‐bHLH‐WD40 (MBW) complex. Plant Signal. Behav. 9, e27522. 24393776 10.4161/psb.27522 PMC4091223

[pbi13213-bib-0026] Li, S.T. and Zachgo, S. (2013) TCP3 interacts with R2R3‐MYB proteins, promotes flavonoid biosynthesis and negatively regulates the auxin response in *Arabidopsis thaliana* . Plant J. 76, 901–913. 24118612 10.1111/tpj.12348

[pbi13213-bib-0027] Lindqvist, K. (1960) Inheritance studies in lettuce. Hereditas, 46, 387–470.

[pbi13213-bib-0028] Liobikas, J. , Skemiene, K. , Trumbeckaite, S. and Borutaite, V. (2016) Anthocyanins in cardioprotection: a path through mitochondria. Pharmacol. Res. 113, 808–815. 27038533 10.1016/j.phrs.2016.03.036

[pbi13213-bib-0029] Lu, H.F. , Lin, T. , Klein, J. , Wang, S.H. , Qi, J.J. , Zhou, Q. , Sun, J.J. *et al*. (2014) QTL‐seq identifies an early flowering QTL located near *Flowering Locus T* in cucumber. Theor. Appl. Genet. 127, 1491–1499. 24845123 10.1007/s00122-014-2313-z

[pbi13213-bib-0030] Matsui, K. , Umemura, Y. and Ohme‐Takagi, M. (2008) AtMYBL2, a protein with a single MYB domain, acts as a negative regulator of anthocyanin biosynthesis in Arabidopsis. Plant J. 55, 954–967. 18532977 10.1111/j.1365-313X.2008.03565.x

[pbi13213-bib-0031] Michelmore, R. , Marsh, E. , Seely, S. and Landry, B. (1987) Transformation of lettuce (*Lactuca Sativa*) mediated by *Agrobacterium tumefaciens* . Plant Cell Rep. 6, 439–442. 24248927 10.1007/BF00272777

[pbi13213-bib-0032] Michelmore, R.W. , Paran, I. and Kesseli, R.V. (1991) Identification of markers linked to disease‐resistance genes by bulked segregant analysis: a rapid method to detect markers in specific genomic regions by using segregating populations. Proc. Natl Acad. Sci. USA, 88, 9828–9832. 1682921 10.1073/pnas.88.21.9828 PMC52814

[pbi13213-bib-0033] Morais, C.A. , de Rosso, V.V. , Estadella, D. and Pisani, L.P. (2016) Anthocyanins as inflammatory modulators and the role of the gut microbiota. J. Nutr. Biochem. 33, 1–7. 27260462 10.1016/j.jnutbio.2015.11.008

[pbi13213-bib-0034] Mulabagal, V. , Ngouajio, M. , Nair, A. , Zhang, Y. , Gottumukkala, A.L. and Nair, M.G. (2010) In vitro evaluation of red and green lettuce (*Lactuca sativa*) for functional food properties. Food Chem. 118, 300–306.

[pbi13213-bib-0035] Oikawa, T. , Maeda, H. , Oguchi, T. , Yamaguchi, T. , Tanabe, N. , Ebana, K. , Yano, M. *et al*. (2015) The birth of a black rice gene and its local spread by introgression. Plant Cell, 27, 2401–2414. 26362607 10.1105/tpc.15.00310 PMC4815089

[pbi13213-bib-0036] Qin, X.X. , Zhang, M.Y. , Han, Y.Y. , Hao, J.H. , Liu, C.J. and Fan, S.X. (2018) Beneficial phytochemicals with anti‐tumor potential revealed through metabolic profiling of new red pigmented lettuces (*Lactuca sativa* L.). Int. J. Mol. Sci. 19, 1165. 29641499 10.3390/ijms19041165 PMC5979491

[pbi13213-bib-0037] Quattrocchio, F. , Wing, J. , van der Woude, K. , Souer, E. , de Vetten, N. , Mol, J. and Koes, R. (1999) Molecular analysis of the *anthocyanin2* gene of petunia and its role in the evolution of flower color. Plant Cell, 11, 1433–1444. 10449578 10.1105/tpc.11.8.1433 PMC144295

[pbi13213-bib-0038] Reyes‐Chin‐Wo, S. , Wang, Z.W. , Yang, X.H. , Kozik, A. , Arikit, S. , Song, C. , Xia, L.F. *et al*. (2017) Genome assembly with in vitro proximity ligation data and whole‐genome triplication in lettuce. Nat. Commun. 8, 14953. 28401891 10.1038/ncomms14953 PMC5394340

[pbi13213-bib-0039] Robinson, R.W. , McCreight, J.D. and Ryder, E.J. (1983) The Genes of Lettuce and Closely Related Species. Plant Breed. Rev. 1, 267–293.

[pbi13213-bib-0040] Singh, V.K. , Khan, A.W. , Jaganathan, D. , Thudi, M. , Roorkiwal, M. , Takagi, H. , Garg, V. *et al*. (2016) QTL‐seq for rapid identification of candidate genes for 100 – seed weight and root/total plant dry weight ratio under rainfed conditions in chickpea. Plant Biotechnol. J. 14, 2110–2119. 27107184 10.1111/pbi.12567 PMC5095801

[pbi13213-bib-0041] Tang, Q.W. , Tian, M.Y. , An, G.H. , Zhang, W.Y. , Chen, J.J. and Yan, C.H. (2017) Rapid identification of the purple stem (*Ps*) gene of Chinese kale (*Brassica oleracea* var. *alboglabra*) in a segregation distortion population by bulked segregant analysis and RNA sequencing. Mol. Breed. 37, 153.

[pbi13213-bib-0042] Thompson, R.C. (1938) Genetic relations of some color factors in lettuce. U.S. Dept. Agr. Tech. Bul. 620.

[pbi13213-bib-0043] Walker, A.R. , Davison, P.A. , Bolognesi‐Winfield, A.C. , James, C.M. , Srinivasan, N. , Blundell, T.L. , Esch, J.J. *et al*. (1999) The *TRANSPARENT TESTA GLABRA1* locus, which regulates trichome differentiation and anthocyanin biosynthesis in Arabidopsis, encodes a WD40 repeat protein. Plant Cell, 11, 1337–1349. 10402433 10.1105/tpc.11.7.1337 PMC144274

[pbi13213-bib-0044] Wang, N. , Liu, Z.Y. , Zhang, Y. , Li, C.Y. and Feng, H. (2018a) Identification and fine mapping of a stay – green gene (*Brnye1*) in pakchoi (*Brassica campestris* L. ssp *chinensis*). Theor. Appl. Genet. 131, 673–684. 29209732 10.1007/s00122-017-3028-8

[pbi13213-bib-0045] Wang, X.F. , An, J.P. , Liu, X. , Su, L. , You, C.X. and Hao, Y.J. (2018b) The nitrate‐responsive protein MdBT2 regulates anthocyanin biosynthesis by interacting with the MdMYB1 transcription factor. Plant Physiol. 178, 890–906. 29807931 10.1104/pp.18.00244 PMC6181044

[pbi13213-bib-0046] Winkel‐Shirley, B. (2002) Biosynthesis of flavonoids and effects of stress. Curr. Opin. Plant Biol. 5, 218–223. 11960739 10.1016/s1369-5266(02)00256-x

[pbi13213-bib-0047] Xu, W.J. , Dubos, C. and Lepiniec, L. (2015) Transcriptional control of flavonoid biosynthesis by MYB‐bHLH‐WDR complexes. Trends Plant Sci. 20, 176–185. 25577424 10.1016/j.tplants.2014.12.001

[pbi13213-bib-0048] Zhang, Y.Z. , Xu, S.Z. , Cheng, Y.W. , Ya, H.Y. and Han, J.M. (2016) Transcriptome analysis and anthocyanin‐related genes in red leaf lettuce. Genet. Mol. Res. 15, gmr7023. 10.4238/gmr.15017023 26909931

[pbi13213-bib-0049] Zhang, L. , Su, W.Q. , Tao, R. , Zhang, W.Y. , Chen, J.J. , Wu, P.Y. , Yan, C.H. *et al*. (2017) RNA sequencing provides insights into the evolution of lettuce and the regulation of flavonoid biosynthesis. Nat. Commun. 8, 2264. 29273740 10.1038/s41467-017-02445-9 PMC5741661

