# Supplementary material for: Characterization of four polymorphic genes controlling red leaf colour in lettuce that have undergone disruptive selection since domestication
Source: Plant Biotechnol J. 2019 Aug 8;18(2):479–90. doi: 10.1111/pbi.13213 (PMC6953203; doi:10.1111/pbi.13213)
Supplement: Supplementary file 1 — Figure S1 Characterization of RLL1. Figure S2 Mapping and functional analysis of the RLL2 gene. Figure S3 Phylogenetic analysis of RLL4 and its homologs. Table S1 Summary of the 145 wild lettuce accessions. Table S2 Primers used in this study. [file PBI-18-479-s001.docx]

**Supplemental Figures and Tables**

**
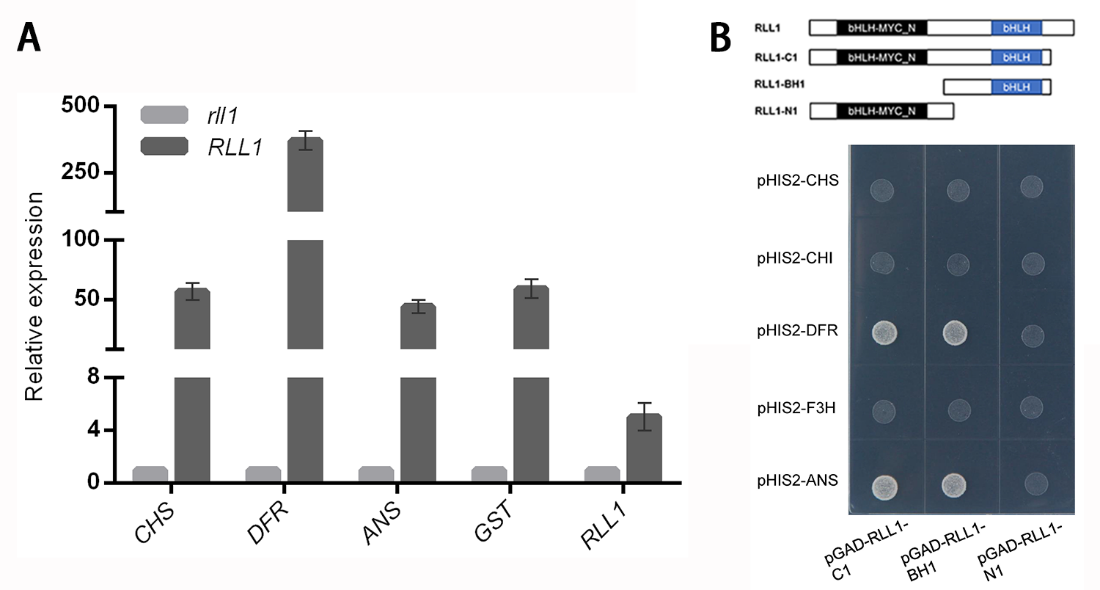
**

**Supplemental Figure S1. Characterization of *RLL1*.** **(A)** Expression of anthocyanin-associated genes in *RLL1* and *rll1*. Gene expression was quantified in the NILs using qRT-PCR. Ubiquitin was used as an internal control. Data are means ± s.e.m (n = 3 biological replicates). **(B)** Y1H assay testing for interactions between the RLL1 protein and the promoter of anthocyanin-associated genes. The names of the genes tested are indicated on the left. The names of the truncated proteins that were used to avoid self-activation are indicated on the left panel.


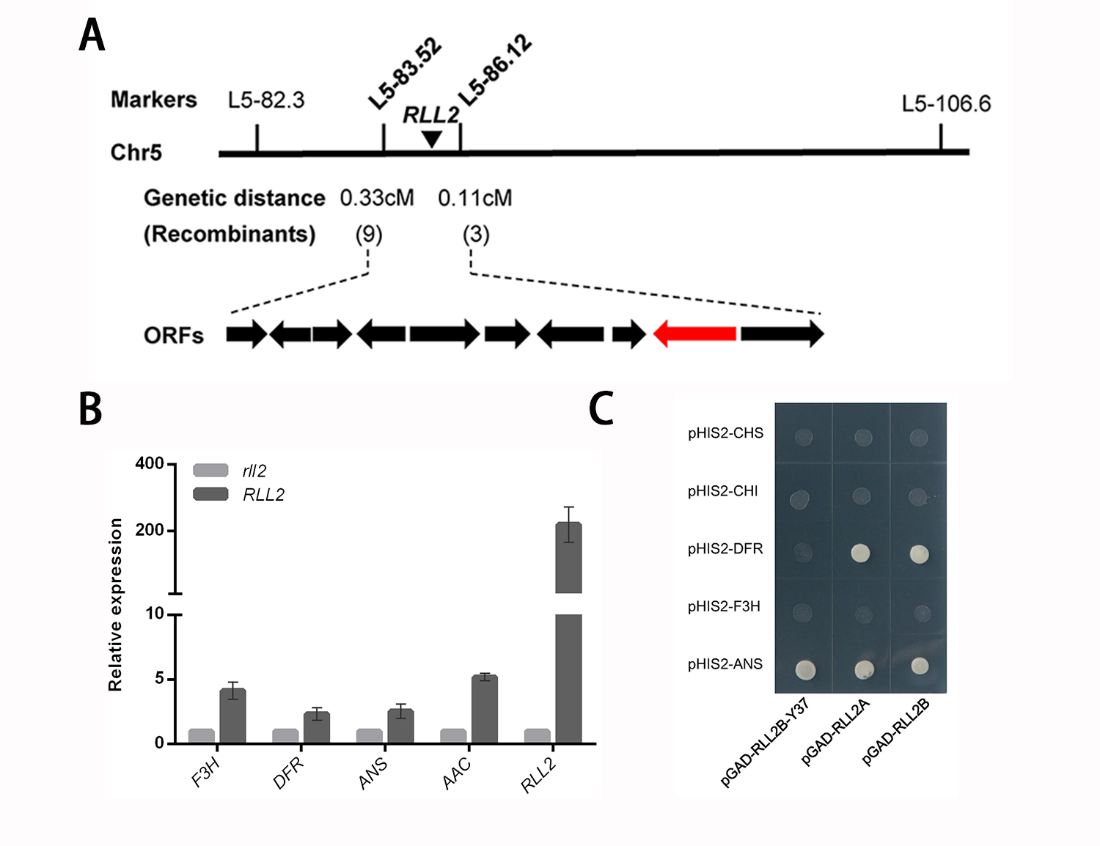


**Supplemental Figure S2. Mapping and functional analysis of the *RLL2* gene.** **(A)** The *RLL2* gene was fine mapped to a region with 10 genes and a MYB-encoding gene (marked red) was identified as the most likely candidate gene. **(B)** The expression of *RLL2* and anthocyanin associated genes. Data are means ± s.e.m (n = 3 biological replicates). (**C**) Y1H shows that RLL2A and RLL2B protein binds to the promoters of the *DFR* and *ANS* genes from the anthocyanin biosynthesis pathway, and RLL2B-Y37 protein binds to the promoters of *ANS* gene.


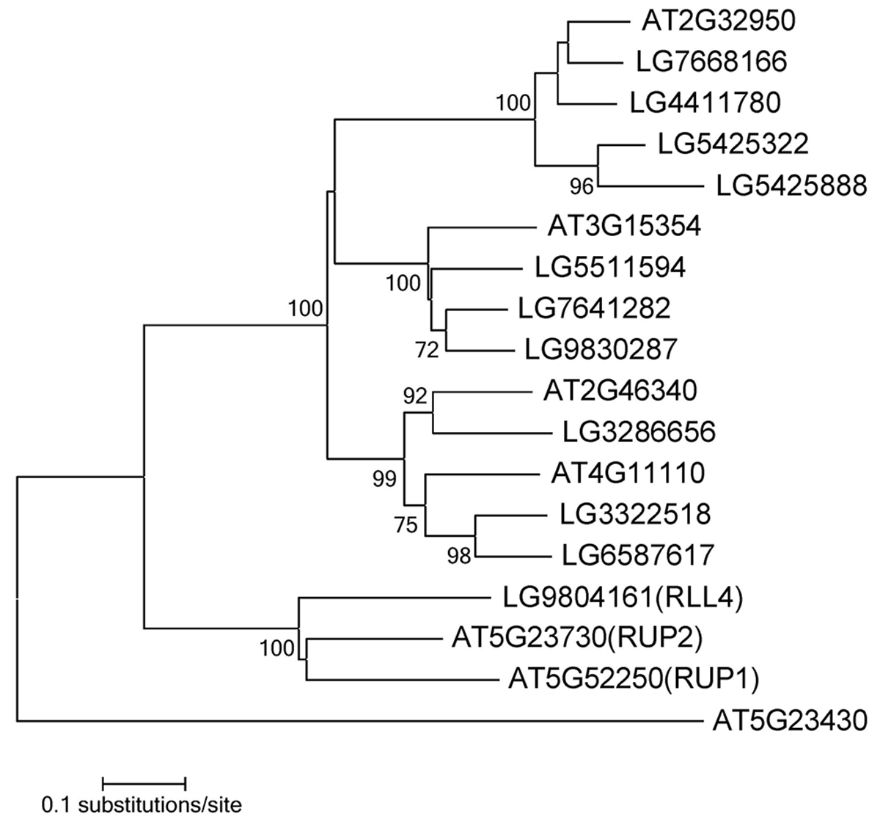


**Supplemental Figure S3. Phylogenetic analysis of RLL4 and its homologs.** The most similar homologs of RLL4 from the genomes of Arabidopsis and lettuce were retrieved to show their phylogenetic relationship with RLL4. Only the conserved WD40 domains were used to construct the neighbor-joining (NJ) tree. Bootstrap values are percentage of 1,000 replicates and values lower than 65 are not shown. RLL4 is obviously orthologous to the RUP1 and RUP2 proteins from Arabidopsis.

**Supplementary Table 1. Summary of the 145 wild lettuce accessions.**

| **PI/CGN #** | **Botanical name** | **Country of origin** |
| --- | --- | --- |
| CGN11317 | *L. serriola* | - |
| CGN05996 | *L. saligna* | - |
| CGN10978 | *L. serriola* | Afghanistan |
| CGN10979 | *L. serriola* | Afghanistan |
| CGN15673 | *L. serriola* | Armenia |
| W6-37147 | *L. serriola* | Armenia |
| CGN15729 | *L. serriola* | Azerbaijan |
| CGN22691 | *L. serriola* | Azerbaijan |
| CGN23091 | *L. serriola* | Azerbaijan |
| CGN13325 | *L. virosa* | Belgium |
| CGN20681 | *L. serriola* | Belgium |
| CGN10939 | *L. serriola* | Bulgaria |
| CGN13369 | *L. serriola* | Bulgaria |
| CC-1 | *L. serriola* | China |
| CC-2 | *L. serriola* | China |
| CC-3 | *L. serriola* | China |
| CC-5 | *L. serriola* | China |
| GY-1 | *L. serriola* | China |
| GY-10 | *L. serriola* | China |
| GY-11 | *L. serriola* | China |
| GY-13 | *L. serriola* | China |
| GY-14 | *L. serriola* | China |
| GY-15 | *L. serriola* | China |
| GY-16 | *L. serriola* | China |
| GY-17 | *L. serriola* | China |
| GY-18 | *L. serriola* | China |
| GY-19 | *L. serriola* | China |
| GY-2 | *L. serriola* | China |
| GY-20 | *L. serriola* | China |
| GY-21 | *L. serriola* | China |
| GY-22 | *L. serriola* | China |
| GY-25 | *L. serriola* | China |
| GY-26 | *L. serriola* | China |
| GY-27 | *L. serriola* | China |
| GY-3 | *L. serriola* | China |
| GY-4 | *L. serriola* | China |
| GY-6 | *L. serriola* | China |
| GY-7 | *L. serriola* | China |
| GY-8 | *L. serriola* | China |
| LT-1 | *L. serriola* | China |
| LT-3 | *L. serriola* | China |
| SHZ-1 | *L. serriola* | China |
| SHZ-2 | *L. serriola* | China |
| SHZ-8 | *L. serriola* | China |
| SHZ-9 | *L. serriola* | China |
| YL-10 | *L. serriola* | China |
| YL-13 | *L. serriola* | China |
| YL-14 | *L. serriola* | China |
| YL-15 | *L. serriola* | China |
| YL-16 | *L. serriola* | China |
| YL-17 | *L. serriola* | China |
| YL-18 | *L. serriola* | China |
| YL-20 | *L. serriola* | China |
| YL-21 | *L. serriola* | China |
| YL-22 | *L. serriola* | China |
| YL-23 | *L. serriola* | China |
| YL-24 | *L. serriola* | China |
| YL-27 | *L. serriola* | China |
| YL-28 | *L. serriola* | China |
| YL-29 | *L. serriola* | China |
| YL-3 | *L. serriola* | China |
| YL-4 | *L. serriola* | China |
| YL-5 | *L. serriola* | China |
| YL-6 | *L. serriola* | China |
| YL-7 | *L. serriola* | China |
| YL-8 | *L. serriola* | China |
| YL-9 | *L. serriola* | China |
| CGN10895 | *L. serriola* | Croatia |
| CGN10896 | *L. serriola* | Croatia |
| CGN10897 | *L. serriola* | Croatia |
| 190906 | *L. serriola* | Czech Republic |
| CGN21380 | *L. serriola* | Denmark |
| CGN21407 | *L. serriola* | Denmark |
| CGN05114 | *L. serriola* | France |
| CGN09392 | *L. serriola* | France |
| CGN23864 | *L. serriola* | France |
| CGN15709 | *L. serriola* | Georgia |
| CGN10878 | *L. serriola* | Germany |
| CGN17418 | *L. serriola* | Germany |
| CGN17419 | *L. serriola* | Germany |
| CGN17429 | *L. serriola* | Germany |
| CGN11335 | *L. serriola* | Greece |
| CGN19051 | *L. serriola* | Greece |
| CGN19052 | *L. serriola* | Greece |
| CGN19059 | *L. serriola* | Greece |
| CGN14230 | *L. serriola* | Hungary |
| CGN14236 | *L. serriola* | Hungary |
| CGN09365 | *L. virosa* | Iran |
| CGN22692 | *L. serriola* | Iran |
| CGN22694 | *L. serriola* | Iran |
| CGN22695 | *L. serriola* | Iran |
| CGN04799 | *L. serriola* | Iraq |
| CGN04800 | *L. serriola* | Iraq |
| CGN05092 | *L. serriola* | Israel |
| CGN09279 | *L. serriola* | Israel |
| 667825 | *L. serriola* | Israel |
| CGN05883 | *L. serriola* | Israel |
| CGN05916 | *L. serriola* | Israel |
| CGN05953 | *L. serriola* | Italy |
| CGN17389 | *L. serriola* | Italy |
| CGN23893 | *L. serriola* | Italy |
| CGN22046 | *L. serriola* | Kyrgyzstan |
| CGN22048 | *L. serriola* | Kyrgyzstan |
| CGN22049 | *L. serriola* | Kyrgyzstan |
| CGN22050 | *L. serriola* | Kyrgyzstan |
| CGN22053 | *L. serriola* | Kyrgyzstan |
| CGN22055 | *L. serriola* | Kyrgyzstan |
| CGN22751 | *L. serriola* | Kyrgyzstan |
| CGN22753 | *L. serriola* | Kyrgyzstan |
| CGN22757 | *L. serriola* | Kyrgyzstan |
| CGN21410 | *L. serriola* | Netherlands |
| Paris | *L. serriola* | Paris |
| CGN21382 | *L. serriola* | Poland |
| CGN23871 | *L. serriola* | Poland |
| CGN23894 | *L. serriola* | Poland |
| CGN09309 | *L. serriola* | Portugal |
| CGN14295 | *L. serriola* | Portugal |
| CGN05075 | *L. serriola* | Romania |
| CGN23855 | *L. serriola* | Romania |
| CGN23904 | *L. serriola* | Romania |
| CGN15731 | *L. serriola* | Russian Federation |
| CGN15733 | *L. serriola* | Russian Federation |
| CGN21379 | *L. serriola* | Slovakia |
| CGN05784 | *L. serriola* | Slovenia |
| CGN13332 | *L. serriola* | Spain |
| CGN13334 | *L. serriola* | Spain |
| CGN13335 | *L. serriola* | Spain |
| CGN21373 | *L. serriola* | Sweden |
| CGN05804 | *L. serriola* | Switzerland |
| CGN10907 | *L. serriola* | Syrian Arab Republic |
| CGN10902 | *L. serriola* | Syrian Arab Republic |
| 491178 | *L. serriola* | Turkey |
| CGN04757 | *L. serriola* | Turkey |
| CGN11331 | *L. serriola* | Turkey |
| CGN11333 | *L. serriola* | Turkey |
| CGN11329 | *L. serriola* | Turkey |
| CGN13330 | *L. saligna* | Turkey |
| CGN23882 | *L. serriola* | Turkey |
| CGN20115 | *L. serriola* | United Kingdom |
| CGN20116 | *L. serriola* | United Kingdom |
| CGN20117 | *L. serriola* | United Kingdom |
| CGN21396 | *L. saligna* | United Kingdom |
| CGN22034 | *L. serriola* | Uzbekistan |
| CGN22037 | *L. serriola* | Uzbekistan |
| CGN22041 | *L. serriola* | Uzbekistan |
| Note: - refers to country unknown | |  |

**Supplementary Table 2. Primers used in this study.**

| **Primer** | **Primer sequence (5' - 3')** | **Purpose** |
| --- | --- | --- |
| L5-86.12-F | GCCCACAAGTCCTCTGGCCT | markers were designed at four loci and were used to screen the F2 population |
| L5-86.12-R | TCGGCTTTCAGACACCACAGAGA |  |
| L5-333.08-F | ACTCGAAAGAAGCACGTTCATTTGGTA |  |
| L5-333.08-R | CCCTGCTTCGTCTGCTAGACACC |  |
| L9-152.76-F | CAACGACGACAAAGGGCCACA |  |
| L9-152.76-R | TCTCCGGGAGCGGTTTCAGTA |  |
| L4-47.92-F | CCTCCTCATAAAGCTCGGGTGCC |  |
| L4-47.92-R | TCGGGAAGTTCTCGGTAAAGGTGC |  |
| L5-335.69-F | CATGAGTAACCCGTGGAGGT | Flanking markers of *RLL1* |
| L5-335.69-R | CGCGTTGTGCAGAAACATCT |  |
| L5-337.92-F | CTCGCCATGAACCGCACCCA |  |
| L5-337.92-R | GCCAATGCATTCCAAGGTGAAGCC |  |
| RLL1-F | ATGGCCGTTGCTCTTGCTCAACCTTAC | amplify the CDS of *RLL1* |
| RLL1-S-R | TCATTGTGTGAGTAGCAACGGAG |  |
| RLL1-Y-R | CTAGTCTCCGCCACCGTGTGATGATG |  |
| L5-83.52-F | TGGGCGGTAGTGGGCAAGGA | Flanking markers of *RLL2* |
| L5-83.52-R | GGCAGGCCCTGTGACACTCG |  |
| L5-86.12-F | GCCCACAAGTCCTCTGGCCT |  |
| L5-86.12-R | TCGGCTTTCAGACACCACAGAGA |  |
| RLL2A-F | ATGACATCGCACAGCCAC | amplify the full length of *RLL2A* |
| RLL2A-R | TCATAGTTGCTGTGAATCCATAG |  |
| RLL2B-F | ATGACATCGCACAGCCACAGTAGTAG | amplify the CDS of *RLL2B* |
| RLL2B-R | TCATAGTTGCTGTGAATCCATAGAG |  |
| RLL2C-F1 | GACATTACAGTTAACAACCG | amplify the second exon of *RLL2C* |
| RLL2C-R1 | AAACCGTAAGTATCACAAGG |  |
| RLL2C-F2 | GTAGCCTACAACTCTTTCTA | amplify the first exon of *RLL2C* |
| RLL2C-R2 | CACGTAGGTGATATGTATGG |  |
| RLL2B-Y37-F | ATGACACCAAGCAGTAGTAATG | amplify the CDS of *RLL2B-Y37* |
| RLL2B-Y37-R | TCATTAAAGCAGGACTCTAGATCA |  |
| L4-49.06-F | ACAACCCCAAACCTGCGGCT | Flanking markers of *RLL3* |
| L4-49.06-R | GCACAACGCCGTCGTCCACT |  |
| L4-49.59-F | GTCGACACGGTGCCGCTGAA |  |
| L4-49.59-R | ACCTTGTTTCGGCAACGACGTA |  |
| RLL3-F | ATGGATAAACGTAGGAGGAGGAAG | amplify the CDS of *RLL3* |
| RLL3-R | TCAAGATTTGACTCTTTTCCTTAAATC |  |
| L9-63.89-F | TCCACCAGCTGCATCCTTTT | Flanking markers of *RLL4* |
| L9-63.89-R | TGCTCGAATGCTTCTTGCTT |  |
| L9-64.25-F | TTCACACAGCCATTGAGCCC |  |
| L9-64.25-R | TGAAGCTTGACATCCGCCAA |  |
| RLL4-F | ATGAAAAACGTCTCATTTCAATC | amplify the full length of *RLL4* |
| RLL4-R | TTAAGTTAACGGTTTTCTTTTTCC |  |
| RLL1-F1 | CAGAATTAGCACAGGAGGAC | Indel marker, used to detect the 5-bp deletion in *RLL1* |
| RLL1-R1 | GAATCTTCTTACGAAGCTGCT |  |
| RLL2-TR-F1 | AGAGGAGACGTTAAAGTTGT | designed at conserved regions and used to analyze the copy numbers in different genotypes |
| RLL2-TR-R2 | GATGAAGTTGATCTCATGCT |  |
| RLL3-dCAPs-F1 | ATTTATATGGTACACATTTCAGAT | dCAPs marker, used to detect the point mutation in *RLL3* |
| RLL3-dCAPs-R1 | TCAAGATTTGACTCTTTTCC |  |
| RLL4-dCAPs-F1 | TAGAGGCAACATTGTTTGACCA | dCAPs marker, used to detect the point mutation in *RLL4* |
| RLL4-dCAPs-R1 | GGTCTCAAGATCGTACTCCGAG |  |
